# Non-traditional Use of HEOR To Identify Host Response Treatments During a Pandemic

**DOI:** 10.36469/001c.24591

**Published:** 2021-06-25

**Authors:** Peter J. Mallow, David S. Fedson

**Affiliations:** 1 Health Services Administration Xavier University, Cincinnati, OH, USA

**Keywords:** heor, host response, covid-19

The traditional use of health economics and outcomes research (HEOR) has been in post-approval (Phase IV) studies of health-care treatments for given indications.^1^ This research is widely used by payors, physicians, providers, and governments to confirm the results of clinical trials, monitor real-world utilization, provide comparisons with similar therapeutic treatments, and detect safety signals. The advent of Coronavirus Disease-2019 (COVID-19) has highlighted the need for and the ability of HEOR to assist in the rapid identification of existing treatments that could be repurposed to treat pandemic patients.

Despite the successful development of several vaccines, it will still take years to vaccinate all of those throughout the world who want to be vaccinated. Unfortunately, anti-viral treatments have been underwhelming.^2^ Further, even if anti-virals prove to be safe and efficacious, they will be expensive and this will limit their use, especially in low-to-middle-income countries. Thus, new approaches are needed for treating COVID-19 patients, especially in non-hospital settings.^3,4^ Physicians have been using pharmaceutical therapeutics off-label from the beginning of the pandemic in an effort to treat COVID-19.^5^ Many of these repurposed drugs target the virus while a few target the host response to infection. Those that target the host response can lessen the effects of illness and allow patients to recover before their host response causes irreparable harm. The near universal availability of electronic health records and claims data in high-income countries provides HEOR researchers with unique opportunities to rapidly study off-label treatments across thousands of patients from different sites of care without relying on the aggregation of single-center case studies, which often take months or years to be published.

One example that highlights the role of HEOR in identifying therapies to treat the host response of COVID-19 is the use of statins. Statins have been shown to reduce endothelial inflammation and may mitigate the host response of COVID-19.^4^ The endothelium is a thin layer of cells that serves as the barrier between the blood and body tissues. It normally regulates vascular permeability and blood coagulation. When inflamed from COVID-19, endothelial cells lose their ability to maintain barrier function and these changes contribute to thrombosis, which can lead to tissue injury and death.^6^

A recent article in JHEOR illustrates the value in identifying promising host response therapies.^7^ COVID-19 patients who received statins in the hospital had significantly reduced mortality. The odds ratio was 0.54 (95% confidence interval [CI] 0.49-0.60; *P*<0.001). Several limitations associated with this finding are common to all observational studies using retrospective claims data. However, the ability to detect a signal that statins may have had a beneficial effect in a study of more than 20 000 COVID-19 patients illustrates the value of non-traditional use of HEOR in identifying potentially effective host response treatments.

Meta-analyses of observational studies have further confirmed the association of in-hospital statin treatment with a reduction in COVID-19 mortality.^8^ A meta-analysis can provide confidence in the evidence from HEOR studies and justify further investigation of statin treatment of the host response to COVID-19. The next step includes randomized controlled trials. As of this writing, nine prospective randomized controlled trials are ongoing to test the efficacy of statins in alleviating the host response to COVID-19, as well as potential anti-viral effects.^3,9^

Hopefully, these prospective clinical studies will demonstrate statin treatment is beneficial in treating COVID-19. If proven effective, widely available generic statins could be used globally with little concern for cost or storage requirements. However, even if the clinical trials do not show a benefit from statin use, it does not negate the use of HEOR to identify existing drugs that could be repurposed for COVID-19 treatment.

As this example has shown, HEOR studies may not be definitive but they represent a good beginning. Subsequent steps such as confirmatory observational studies, meta-analyses, and prospective clinical studies are still required. However, HEOR can reduce the time required for identifying a promising treatment and determining its efficacy. This time may save lives.

In order for HEOR to fulfill this potential several things must be in place. First, there must be an *a priori* identified mechanism of action for the therapy being investigated. In the case of Mallow et al.^7^, several experimental and clinical studies had shown that those with COVID-19 suffer from endothehial dysfunction, which may be amenable to statins.^6^ Second, timely access to electronic health records and claims data from multiple sites is critical. In the last few years, databases, such as IBM MarketScan Research Databases and Premier Healthcare Database, have become timelier with increased interoperability and automated adjudication processes. Third, there needs to be robust yet timely peer-review of the findings to provide researchers with the evidence necessary to conduct meta-analyses of potentially useful repurposed drugs and support the development of prospective clinical trials.

COVID-19 has united the world’s researchers and has demanded innovative thinking to address this pandemic. HEOR has been no different and can play a powerful role identifying treatments to address the host response, in addition to COVID-19. The need is to move past the traditional uses of HEOR and contribute to identification of existing drugs that can be repurposed to treat the host response. Failure to do so is a sin of omission.

## DECLARATIONS

Conflicts and Financial Disclosures: The authors did not receive any financial payment in connection with this work. Peter Mallow reports consulting relationships with CTI Clinical Trial and Consulting, Inc., Cardinal Health Inc., Health Clarity Solutions, LLC, and Abiomed Inc. David Fedson reports no conflicts of interest.
